# Sodium nitrite as a corrosion inhibitor of copper in simulated cooling water

**DOI:** 10.1038/s41598-021-87858-9

**Published:** 2021-04-16

**Authors:** Marziya Rizvi, Husnu Gerengi, Savas Kaya, Ilyas Uygur, Mesut Yıldız, Ibrahim Sarıoglu, Zafer Cingiz, Michal Mielniczek, Brahim El Ibrahimi

**Affiliations:** 1 Corrosion Research Laboratory, Department of Mechanical Engineering, Faculty of Engineering, Duzce University, Duzce, Turkey; 2 Department of Pharmacy, Health Services Vocational School, Sivas Cumhuriyet University, 58140 Sivas, Turkey; 3 Department of Electricity and Energy, Duzce Vocational High School, Duzce University, Duzce, Turkey; 4 Corrosion and Materials Engineering, Department of Electrochemistry, Faculty of Chemistry, Gdansk University of Technology, 11/12 Narutowicza, 80-233 Gdańsk, Poland; 5 Applied Chemistry-Physics Team, Materials and Environment Laboratory, Faculty of Sciences, Ibn Zohr University, B.P.8106, Cité Dakhla, Agadir, Morocco

**Keywords:** Chemistry, Materials science

## Abstract

The corrosion inhibition behavior of sodium nitrite (NaNO_2_) towards pure copper (99.95%) in simulated cooling water (SCW) was investigated by means of electrochemical impedance spectroscopy (EIS) and dynamic electrochemical impedance spectroscopy (DEIS). NaNO_2_ interferes with metal dissolution and reduce the corrosion rate through the formation or maintenance of inhibitive film on the metal surface. Surface morphologies illustrated that the surface homogeneity increased on adding sodium nitrite. Sodium nitrite’s adsorption on copper surface followed the modified form of Langmuir, Freundlich and Frumkin isotherms. Physiosorption mode was involved in the corrosion protection. Electrochemical results revealed an corrosion resistance of copper increases on increasing the inhibitor concentration. The DEIS results indicated that copper corrosion mechanism could be hindered by 50% even after interval of 24 h by optimum concentration of sodium nitrite. The maximum inhibition was achieved with 2000 ppm of NaNO_2_. With this concentration, inhibition efficiency of up to 61.8% was achievable.

## Introduction

The industrial cost of corrosion worldwide has been calculated to be 3–4% of the gross national product (GNP). Additionally, 20% of this loss is evitable if suitable methods are used for corrosion protection^1,2^. The major motive for investigating corrosion is to find the techniques and methods to minimize or control this problem.

Copper has been widely used industrially because of its corrosion resistance, electrical, thermal and mechanical properties^3,4^. It is also an integral part in the fabrication of wire, pipelines and sheets in electronic factories, marine stations, power houses, cooling towers and heat exchangers^5,6^. Copper is considerably corrosion resistant to the atmospheric corrosive agents. Even in some of corrosive electrolytes, it easily protects itself from degradation by forming a passive oxide film which is non-conductive in nature^7,8^. However, pitting corrosion is accelerated on proximity of the O_2_ and some halide likes chloride. Scaling caused by carbonates and sulfates also accelerates the corrosion if the electrolyte seeps below the deposited layer^9,10^. This behavior protects copper from excessive damage, but it is impairing to the system performance and efficiency of the equipment which is fabricated using this metal^11^. The equipments like heat exchangers, boilers, pipelines, coils, electrical boards and circuits constructed from copper are generally in close proximity to the corrosive environment in the desalination systems or petroleum pipelines^12–17^ etc. Moreover, tidal/sea waves forming the dry and wet cycles enhance this corrosion as the passive layer containment on the copper surface is difficult in such a situation^18–21^. Increasing research publications related to copper protection by corrosion inhibitors indicate the importance of this industrial problem^22,23^. Although considered a resistant metal, copper may still suffer from corrosion in aqueous conditions by harsh ions like halides, sulfates, etc. Another hindrance to its application is fouling, which is caused by colonies of microorganisms thriving over a long time in the neutral or basic cooling water systems^24^. Efficiency and longevity of cooling coils/pipelines depend on the protection of copper surface which should be devoid of pits and accumulated toxic wastes after degradation.

This research article provides important insights on the corrosion susceptibility of copper in simulated cooling water (SCW) which is an integral part of industrial units like boilers, condensers, heat exchangers, pipelines, economizers, etc. Extreme values of pH may lead to corrosion of any metal, but the pH of simulated cooling water lies in a neutral range. Although the pH of the prepared SCW was recorded as 7.07 initially, on dissolving the maximum concentration of inhibitor, it increased to 7.90. Copper is fairly inert metal, however aggressive ions in SCW like chlorides, sulfates, carbonates and bicarbonates can cause copper to degrade and corrode as shown in the following equations.

1 $${\text{Anodic}}\,{\text{Dissolution}}:2{\text{Cu}} \to {\text{Cu}}^{2 + } + 2{\text{e}}^{ - }$$

2 $${\text{Cu}} + {\text{Cl}}^{ - } \leftrightarrow {\text{CuCl}} + {\text{e}}^{ - }$$

The partially soluble cuprous chloride in dilute NaCl reacts to form cuprite or cuprous oxide (Cu_2_O). This cuprous oxide, in the presence of dissolved salts in water, oxidized to cupric hydroxide (Cu(OH)_2_). The dissolved salts in SCW not only lead to corrosion but also scaling and depositions. Normally copper in cooling systems tends to form a protective passive layer of Cu_2_O which later on evolves to CuO/Cu(OH)_2_ which can be confirmed by checking the potential and the pH of electrochemical process in the E-pH diagram of aqueous corrosion of copper^25,26^. Higher temperatures lead to conversion of Cu (OH)_2_ to CuO completely, but as the analysis is done at room temperature, a predominant presence of Cu(OH)_2_ is expected in the passive layer.

Chloride is particularly corrosive to copper, even at basic values of pH. Chloride and sulfates are well known to antagonistically accelerate the degradation of copper. The small aggressive anions migrate through the film to the areas of high positive charge density. Substitution of monovalent chloride species for divalent charged oxygen species might occur leading to the release of Cu^2+^ ions in the bulk solution (corrosion). Calcium and magnesium along with the bicarbonate ions present in SCW protect the copper by forming a deposited film, however localized corrosion may still occur beneath these deposited layers. Hence pitting corrosion is expected to occur profusely on the copper metal in such scenarios.

In order to tackle copper corrosion, corrosion inhibitors are the most convenient and economic options. The role of inhibitor to suppress the anodic or cathodic reactions or both of them, determine the type of corrosion inhibitor it might be. If the addition of corrosion inhibitor to corrosive environment reduces anodic dissolution of copper, the corrosion inhibitor provides anodic inhibition. Corrosion inhibitors, including inorganic or organic compounds like azoles, amines and amino acids effectively protect copper and copper alloys. This action is associated with the chelation effect of the functional groups in them or the formation of impermeable barrier between the inhibitor and the copper, preventing its dissolution. Also, presence of vacant d orbitals in copper atom leads to coordinate interactions with heteroatom such as nitrogen, sulfur and oxygen or π-interaction amidst aromatic rings via the heterocyclic section of the inhibitor. Many organic and inorganic substances have been utilized as corrosion inhibitors of copper in acidic, alkaline and neutral media. While the chromates which increase the corrosion rate by increasing the rate of cathodic reactions, have been rejected because of their toxicity, the tetraborates and molybedates have been observed to form instable protective films on copper surface. The benzotriazoles act by formation of a protective monolayer or multilayer film in presence of oxidants or by anodic polarization. For alkaline and neutral mediums, benzotriazoles have given impressively high corrosion protection capacities yet their non-biodegradability poses a danger of flow of non-biodegradable substances in high quantities in general waterways^27^. Sodium is a neutral species whose effect is non-discernable in cooling water systems’ corrosion. In this research article sodium nitrite has been utilized to check its effect on corrosion of copper in SCW. It is hypothesized and proved that nitrite will suppress the anodic dissolution of copper^28^. The authors in the current research article have tried to explain the exact function of sodium nitrite in the protection of copper in SCW. Sodium nitrite is nontoxic and ecofriendly compound used as a food preservative. It is a well-known fact that the corrosion inhibitors with heteroatoms like ‘N’, tend to adsorb on the surface to form inhibitive film as a barrier between electrolytic solution and metal to stop the corrosion reaction from occurring^29^. The inhibition mechanism of nitrite ion described by previous researchers shows that it starts by conversion of nitrite ions to nitrous oxide and oxygen ions as follows.

3 $$2{\text{NO}}_{2}^{ - } + 4{\text{e}}^{ - } \to {\text{N}}_{2} {\text{O}} + 3{\text{O}}^{2 - }$$

These O^2−^ ions oxidize cuprous ions to cupric. In an alkaline solution, stable passive film of CuO/Cu (OH) _2_ forms on the metal surface^30^.

## Results

### EIS measurements

**Figure 1:**
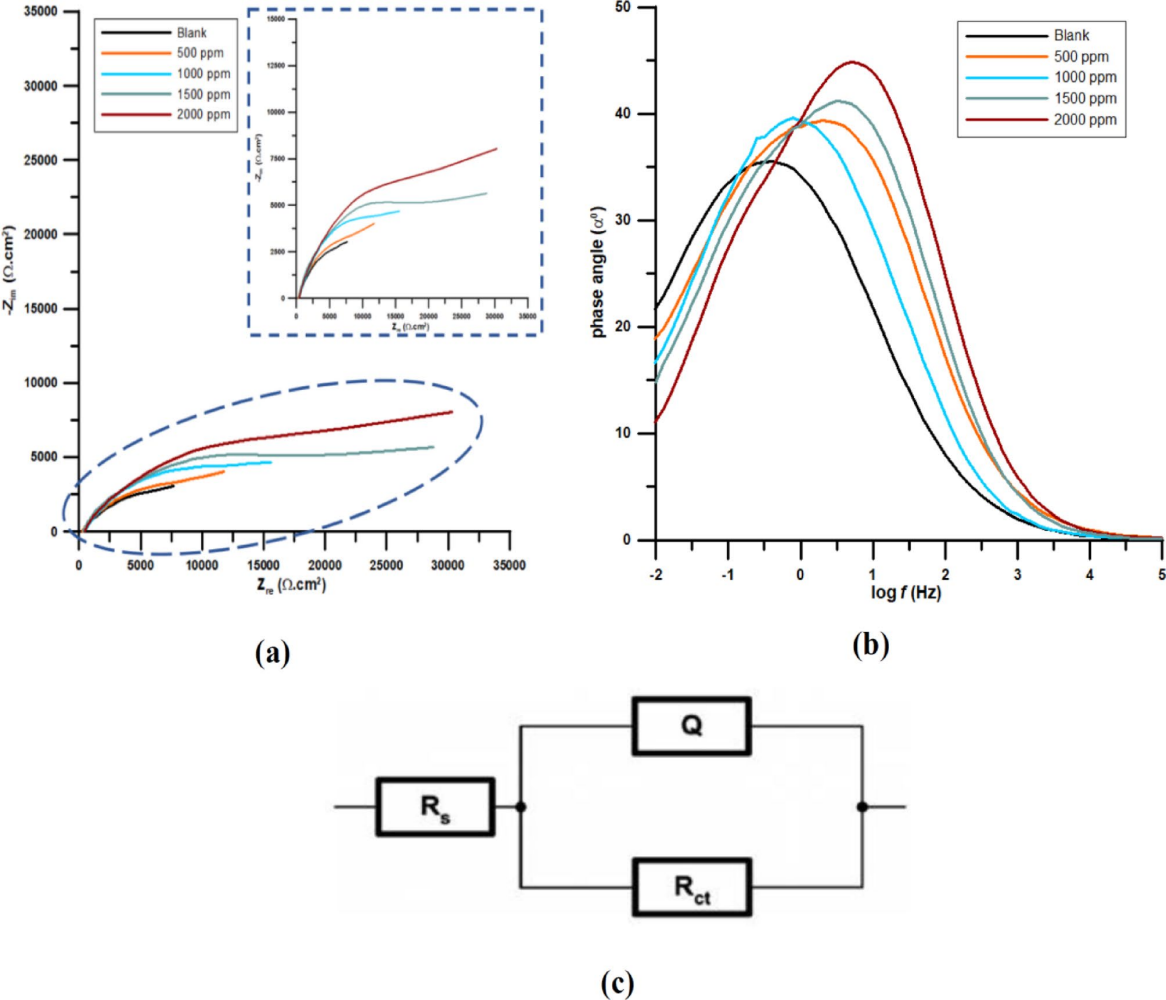
(**a**) Nyquist and (**b**) bode graphs of copper samples in SCW inhibited by different concentrations of NaNO_2_ (**c**) Equivalent circuit model applied for electrochemical analysis.

Impedance spectroscopy is a common practice carried out to estimate the resistance between the interface of a metal and a corrosive medium. Figure 1a shows the EIS data of copper samples subjected to the various concentrations of NaNO_2_ in SCW. The Nyquist plots indicate the capacitive behavior of the spectra which means that the corrosion process of the copper electrode includes charge transfer resistance. The diameter of capacitive semicircle increased with addition of NaNO_2_, implying that the corrosion protective behavior of copper was improved on increasing the inhibitor concentration. The Bode phase plots showed a single constant in the impedance spectrum which was again generally associated with charge transfer process (Fig. 1b). The EIS graphs indicated that the copper surface was covered by a thin film, a passive layer which prevented the attack of the corrosive ions present in SCW. The phase angle diagrams also displayed that maximum phase angle was increased each time the concentration of NaNO_2_ increased in the simulated cooling water. This proves again that addition of NaNO_2_ was beneficial to the growth of inhibitive barrier.

This might be indicative of the fact that the passive layer of oxides or the thin film of Cu^2+^/inhibitor complex covering the copper surface thickens or improve with increasing inhibitor concentration. The same can be understood by the values of *Y*_o_ associated with growth of double layer capacitance. Although the values of *R*_ct_ increase on adding increasing its concentration, an increase in the values of *R*_s_ is also noted. Usually film forming inhibitors get adsorbed to the metal and *R*_s_ tends to decrease. In case of NaNO_2_, it is generally considered anodic inhibitor which helps in forming the thin protective layer of oxide on the copper surface^30^. This implies that due to this oxide barrier on surface, the ion transfer between copper and bulk solution decreases each time the inhibitor concentration is increased that results in increasing *R*_s_. The impedance data is enlisted in Table 1. The impedance values were accurately fitted to *R*(*QR*) circuit and the *χ*^2^ values of 10^−3^ were obtained to ensure the correctness of the this fit (Fig. 1c). *R*_s_ is the non-compensated solution resistance, *R*_ct_ is the charge transfer resistance and *Q* is the constant phase element, CPE used in place of ‘*C*_dl_’ double layer capacitance to account for the non-ideal behavior of the working electrode^31^. ‘n’ is the quantification of the capacitor’s heterogeneity whose value may be in the range of 0 < n < 1. Table 1 displays improvement in surface homogeneity with increasing inhibitor concentration.

**Table 1:** EIS parameters for copper samples in SCW inhibited by different concentrations of NaNO_2_ at room temperature.

Concentration	*R*_s_ (Ω cm^2^)	*Y*_o_ × 10^−4^ (µΩs^n^ m^−2^)	*n*	*R*_ct_ (Ω cm^2^)	*IE* (%)	*χ*^2^ × 10^−3^
Blank	279.4	4.4	0.56	9250	0	0.7
500 ppm	178.8	2.6	0.57	11,500	19.5	0.2
1000 ppm	266.7	1.8	0.59	14,660	36.9	1.3
1500 ppm	349.7	1.1	0.60	20,370	54.5	5.3
2000 ppm	367.4	0.9	0.61	24,230	61.8	6.6

Table 1 distinctly specifies that the values of *R*_ct_ increased with increasing NaNO_2_ concentration. *R*_ct_ values obtained for uninhibited solution are resultant of primarily adsorbed water molecules and other ions on the metal/electrolyte interface, which were later displaced by inhibitor species. A potential of 0.2 V/Ag/AgCl corresponding to pH value of 7.9 might be attributed to the initial formation of Cu_2_O, which evolved to CuO/Cu (OH) _2_ at later stages in uninhibited electrolytic solution. However it can also change to Cu^2+^/Inhibitor in the inhibited solution^26^. At the maximum concentration, NaNO_2_ is 61.8% efficient inhibitor according to EIS.

### DEIS measurements

**Figure 2:**
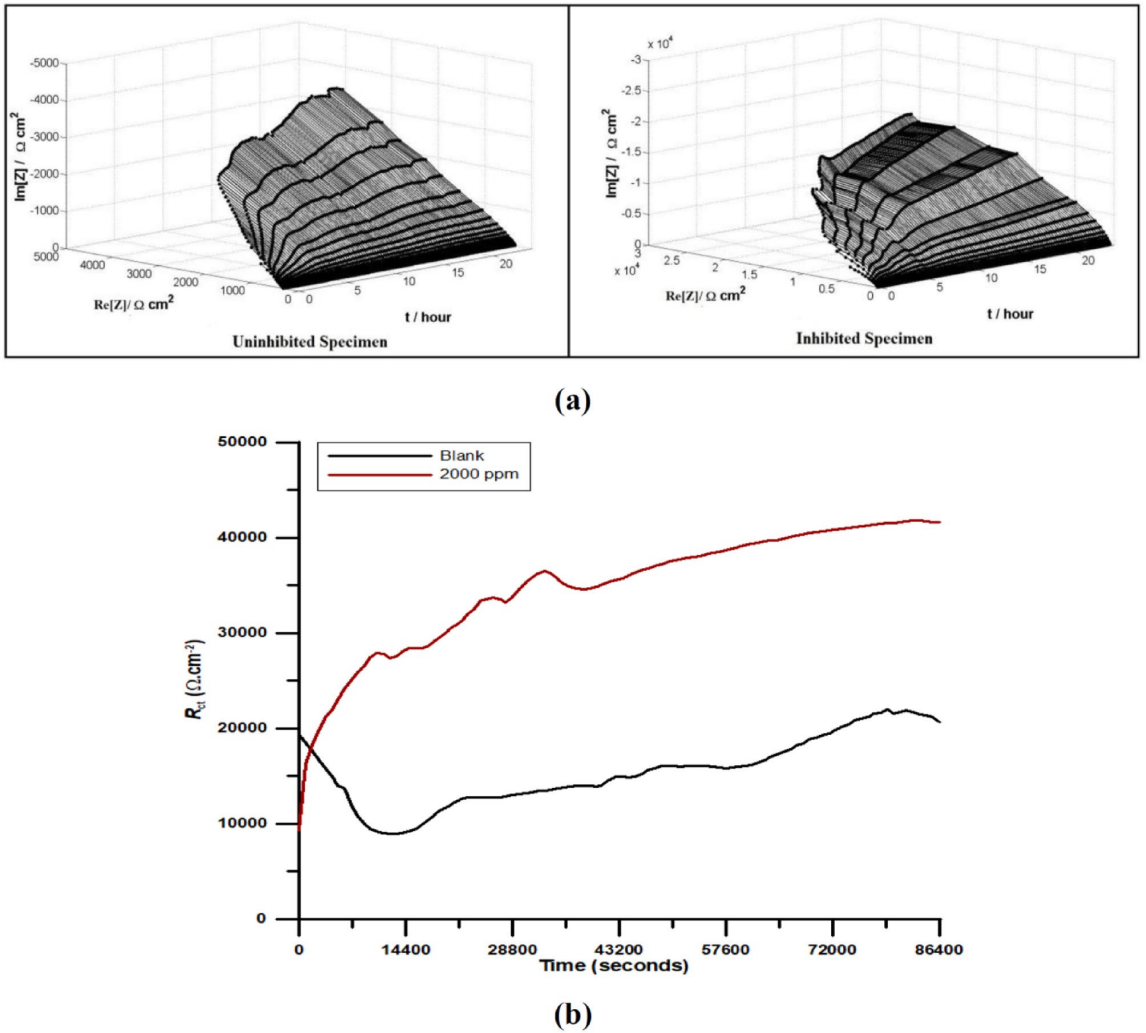
(**a**) DEIS spectra of copper specimens immersed in SCW for 24 h, uninhibited and inhibited by 2000 ppm NaNO_2_ (**b**) *R*_ct_ versus time plot for copper specimen immersed in SCW in inhibited and uninhibited state for 24 h.

DEIS analysis at different time intervals to get an insight for tracking the changes in the adsorbed inhibitor film of nitrite on the copper surface in SCW was carried out up to 24 h of sample immersion. Figure 2a represents the DEIS spectra of 24 h for copper specimens tested at room temperature after attaining a stable open circuit potential (OCP).

In order to gain better understanding of the dissolution pattern of formation of Cu^2+^ ions on the copper surface and prevention of this corrosive reaction by sodium nitrite in SCW the DEIS experiments were conducted for all the environments at different time ranges of 2 h , 6 h, 12 h and 24 h, respectively. It was observed that *R*_ct_ improved with time indicating better performance of inhibitor with increase in its concentration. The equivalent circuit used in the DEIS analysis was similar to EIS analysis (Fig. 1c). As usual, the best fit was determined based on the low values of *χ*^2^ in the range of 10^−3^. The DEIS parameters thus obtained have been given in Table 2. The influence of the addition of the maximum inhibitive concentration of sodium nitrite to SCW can be clearly understood by looking at the charge transfer versus time plot in Fig. 2b.

**Table 2:** Analysis of DEIS Nyquist chart for copper specimen immersed in SCW in inhibited and uninhibited state at time interval of 2 h, 6 h and 24 h.

Time interval/concentration	First spectra		2 h		6 h		12 h		24 h	
	*Y*_o_	*R*_ct_	*Y*_o_	*R*_ct_	*Y*_o_	*R*_ct_	*Y*_o_	*R*_ct_	*Y*_o_	*R*_ct_
Blank	951.7	19,406	315.8	11,532	156.1	12,620	133.6	14,930	112.3	20,730
2000 ppm	97.7	9297	32.09	25,070	17.67	31,200	11.47	35,770	8.09	41,610
% IE	–	–	–	54.01	–	59.55	–	58.26	–	50.18

Unit for *R*_ct is_ (Ω cm^2^) and *Y*_o_ is (µΩs^n^ m^−2^).

The values of *R*_ct_ after 2 h interval with DEIS coincide with EIS result of maximum inhibitor concentration which indicated the consistency of the tests performed. In the case of the uninhibited solution it was observed that *R*_ct_ dips for initial 0 to 14,400 s which could be associated with initial attack of the aggressive ions in the SCW. After 14,400 s a very gradual constant increase in the values of *R*_ct_ was observed which was resistant to the aggressive solution. In the copper specimen inhibited by 2000 ppm of sodium nitrite in SCW, there was no dip in *R*_ct_ values which is due to the inhibition offered by nitrite ions and prevention of dissolution of copper as well as prevention of chloride ions in destroying the passive layer of oxide which formed on the copper surface in aqueous solutions. However, the charge transfer value for the first spectra obtained, immediately after immersion in inhibited solution was lower than that of the blank SCW solution.

The previous researches conducted on the behavior of nitrite ion towards copper and its oxides in different media have concluded that the growth of resistance of initially formed Cu_2_O on the copper surface is hindered by the nitrite ion, however after some time when the Cu_2_O evolves into Cu_2_O + CuO/Cu(OH)_2_, the nitrite ions starts assisting in growth of resistance offered by oxide film by reacting with Cu(OH)_2_^25,26^. In the absence of nitrite ions in the uninhibited solution the growing film of Cu_2_O initially formed on copper specimen exhibited some resistance, along with attack of aggressive ions which displaced oxygen and lead to anodic dissolution. However interestingly, when inhibitor containing nitrite ion was added to the blank SCW solution, it hindered the growth of cuprous oxide layer on the surface which led to ease of attack on SCW ions on the surface and corrosion. Hence, the *R*_ct_ began to evolve from a lower value in the first spectra, than that observed in blank solution (Table 2). However exactly after 14,400 s the development of copper (II) hydroxide along with cupric oxide favoured the corrosion protection by formation of copper nitrite on the surface. This led to the elevation of *R*_ct_ values higher than in blank solution after 2 h and a steady inhibition efficiency in the range of 50–59% was observed over a period of 24 h. At 2 h interval 54% IE was achieved which might be due to acceleration of the growth of passive oxide layer by nitrite ion. At 6 h IE attains a maximum of 59.5%. At 12 h the IE depleted slightly to 58% and further reduced to 50.5% at 24 h interval. Generally it was expected that nitrite ion would keep assisting the growth of passive layer and IE would maintain an increasing trend till 24 h. This lowering of IE suggested that nitrite ion was itself involved in some reaction on the copper surface. If the process of physiosorption is involved in such association, after a set period of time the decline in IE could be explained by the mode of adsorption involved which will be discussed in the following section. Note worthily, after 14,400 s the nitrite ion interferes more with the anodic dissolution of copper and higher *R*_ct_ is obtained compared to the blank SCW solution. The results of this analysis suggested that sodium nitrite was effective in prevention of copper corrosion in SCW.

### Adsorption isotherms

The isotherms involved in depicting the process of adsorption of nitrite ion on the copper surface provided better understanding of the corrosion inhibition in the current study. The adsorption process tends to obey modified Langmuir adsorption isotherm or El-Awady isotherm, Freundlich adsorption isotherm and Frumkin adsorption isotherm based on the values of correlation “*R*^2^” which should be close to unity. The adsorption parameters thus obtained have been enlisted in Table 3.

**Table 3:** Adsorption values obtained for copper specimen in SCW inhibited by NaNO_2_ for 2 h at room temperature.

Adsorption isotherms	Slope	Inercept	*R*^2^	*K*_ads_	*ΔG*_*ads*_ kJ mol^−1^	Interaction parameter
El- Awady	0.7091	3.1436	0.9944	27,108.34	− 24.86	1/y = 1.4
Freundlich	1.1532	3.5098	0.9897	3962.78	− 20.18	n = 1.1
Frumkin	0.7477	5.9139	0.9755	368.97	− 14.39	a = 0.4

**Figure 3:**
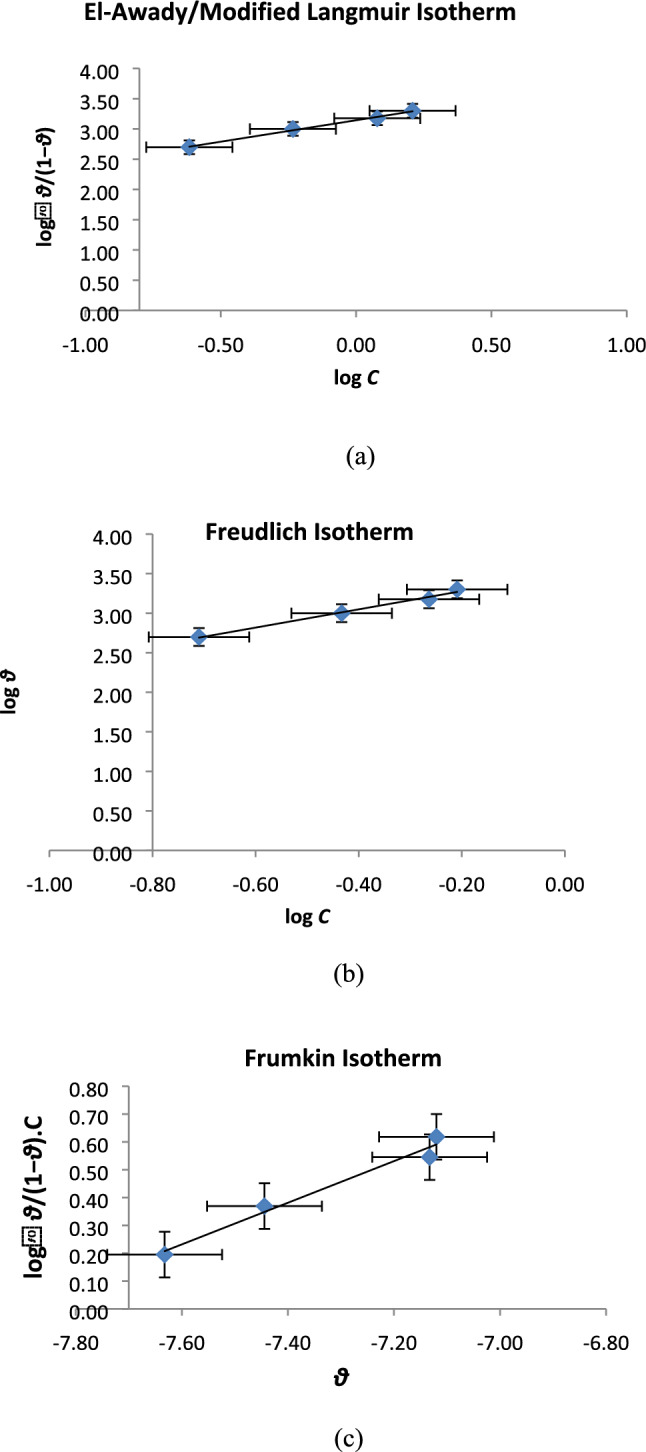
Adsorption isotherms for copper specimen immersed in SCW inhibited by 2000 ppm of NaNO_2_ (**a**) El-Awady Adsorption Isotherm, (**b**) Freundlich Isotherm and (**c**) Frumkin Isotherm.

The experimental data were best suited to modified Langmuir isotherm which is generally called El-Awady isotherm (Fig. 3a)^32^. El-Awady isotherm is described by

4 $$\log \left( {\frac{\uptheta }{{1 -\uptheta }}} \right) = \log K + y\log C$$

where *y* is number of inhibitor molecules accommodated in unit active site, ‘$\theta$’ the surface coverage, ‘*C*’ the concentration and ‘*K*_*ads*_*’* the constant related to the adsorptive equilibrium constant.

5 $$K_{ads} = K^{1/y}$$

When this 1/*y* is below 1 it suggests multilayer adsorption. Currently, the values of 1/*y* obtained were more than one (1/y = 1.4) which meant that each inhibiting molecule was attached to more than one active site on the copper surface. This might be because of the presence of two hydroxide or two nitrite ions in association with the anodic site. The adsorptive equilibrium constant ‘*K*_ads_’ (obtained from the intercept of the graphs of adsorption isotherms) helped in the derivation of adsorption free energy ‘Δ*G*_ads_’ as per following equation:

6 $$K_{ads} = \frac{1}{{C_{{H_{2} O}} }}exp \frac{{ - \Delta G_{ads} }}{RT}$$

where the water molecules concentration is 1000 gm/L, *‘R’* is the universal gas constant (R = 8.314 J K^−1^ mol^−1^) and *T* delineate the absolute temperature (*K*) which is room temperature in the current analysis.

Freundlich isotherm (Fig. 3b) suggests the adsorptions and interactions according to the following equations

7 $$\theta = KC^{n}$$

8 $$\log \theta = \frac{1}{n}log C + \log K_{ads}$$

where 1/*n* = 1.15 is a constant depending on the nature of the adsorbed molecule, *K*_ads_ (3.2 × 10^3^ ppm^−1^ )is the adsorption–desorption constant which displays interaction strength of the adsorbed layer. In fact, when its value is near zero, it means non homogenous surface; whereas, a value below unity means a strong adsorption (chemisorption), similarly, above unity it is indicative of moderate adsorption (comprehensive/mixed). The value of 1/n was slightly more than unity which suggested moderate physiosorption on the copper surface The correlation coefficient for Freundlich isotherm was 0.98^33^.

The Frumkin adsorption isotherm (Fig. 3c) assumes copper surface as heterogeneous and interaction between the adsorbed molecules as per following equation:

9 $$log\frac{\theta }{{\left( {1 - \theta } \right) \cdot C}} = K_{ads} Ce^{{2{{\varvec{\upalpha}}}{\varvec{\theta}}}}$$

10 $$log\frac{\theta }{{\left( {1 - \theta } \right) \cdot C}} = 2.303 log K_{ads} + 2{\upalpha }\theta$$

α (0.37) is related to the molecular interaction in adsorbed layer.where α is the lateral interaction parameter that describes the interaction in the adsorbed layer. The molecular interaction parameter can be assumed positive or negative: when α < 0, there is repulsion in the adsorbed layer, and otherwise attraction^34^.

Negative values of **ΔG**_ads_ meant a spontaneous adsorption. When the values of (**ΔG**_ads_) are up to − 20 kJ mol^−1^, they suggest electrostatic interactions between the charged molecules and the charged metal (physisorption) and when these values are around − 40 kJ mol^−1^, they suggest chemisorption which is as a result of sharing or transfer of electrons from inhibitor molecules to the metal surface to form a coordinate bond (chemisorption)^35^. The values of **ΔG**_ads_ listed in Table 3 were all negative which suggested a spontaneous adsorption of inhibitor on copper surface. The range of the values suggested the predominant role of physiosorption, which implies a minor possibility of formation of a complex on the metal substrate. However El-Awady isotherm suggested the role of comprehensive type of adsorption which might occur in the presence of two heteroatoms N and O directly involved in the protection of copper surface. Summarizing, from the adsorption analysis it was suggested that more than one species of inhibitor was associated with each active site on the copper surface and the adsorbed species were interacting with each other. After 2 h CuO/Cu(OH)_2_ was formed on the surface of copper. There is also a possibility of it undergoing a displacement reaction for nitrite ion to get adsorbed on the copper surface.

### Surface analysis

Surface analysis led to important inferences about the adsorption of NaNO_2_ on copper surface in SCW. The improvement of surface properties, quantitative and qualitative judgment of the molecules adsorbed on the copper specimens could only be understood through surface analysis. One of the important objectives of the study of surface composition of inhibited specimen was to know whether the adsorption is solely dependent on oxygen or nitrogen is also associated with the copper surface.

#### AFM

**Figure 4:**
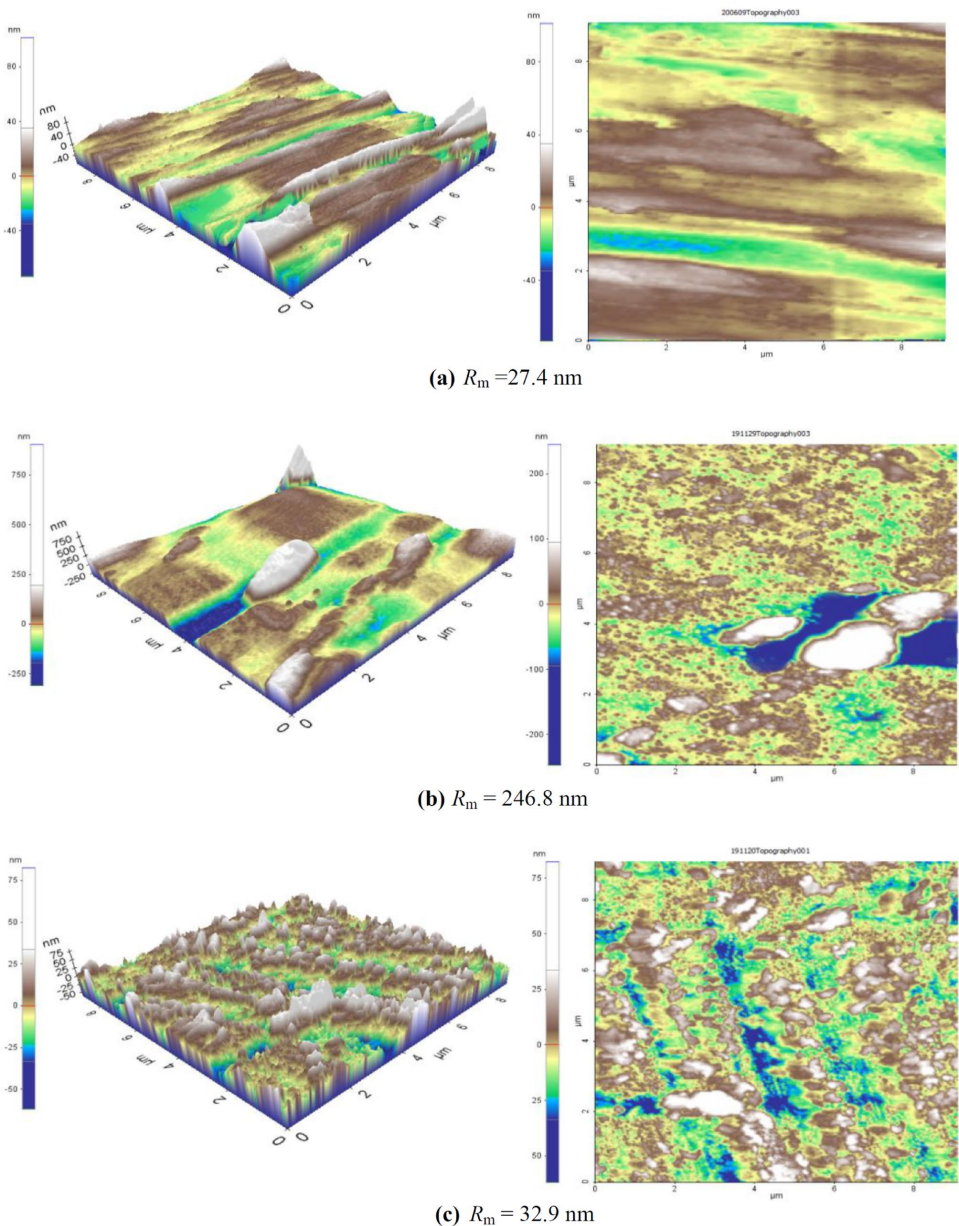
AFM images of (**a**) polished copper specimen, (**b**) copper specimen corroded in SCW and (**c**) copper specimen in SCW protected by 2000 ppm NaNO_2_.

To judge the efficiency and role of NaNO_2_ in corrosion prevention and maintaining the homogeneity of the copper surface when immersed in SCW, 3D and 2D images obtained by AFM were observed for each specimen. The freshly polished copper specimen had a mean roughness factor *R*_m_ of 27.4 nm (Fig. 4a). When this polished specimen was immersed in SCW for 2 h, the roughness was degraded to *R*_m_ = 246.8 nm (Fig. 4b). The abrasion lines apparent in the polished specimen were dissolved in the specimen immersed in SCW.

The corroded specimen in the uninhibited solution, also displayed blue colored regions in 2D view which might be attributed to the pitting caused by chloride ions (Fig. 4a). According to the scale of the images, the blue regions depicted are the areas where the metal was been removed by corrosion. The localized corrosion is very clearly evident in the corroded specimen (Fig. 4b). The roughness of the specimen was less when it was immersed in a solution of SCW inhibited by 2000 ppm of NaNO_2_, apparent reduction of blue regions was observed. The mean roughness improved to *R*_m_ = 32.9 nm (Fig. 4c). Apparently few blue regions (pits) are seen in the 2D image of inhibited specimen in Fig. 4c, which suggests less pitting and low corrosion. Addition of sodium nitrite clearly improved the surface roughness of the copper specimen was very apparent in the AFM images.

#### SEM/EDX

**Figure 5:**
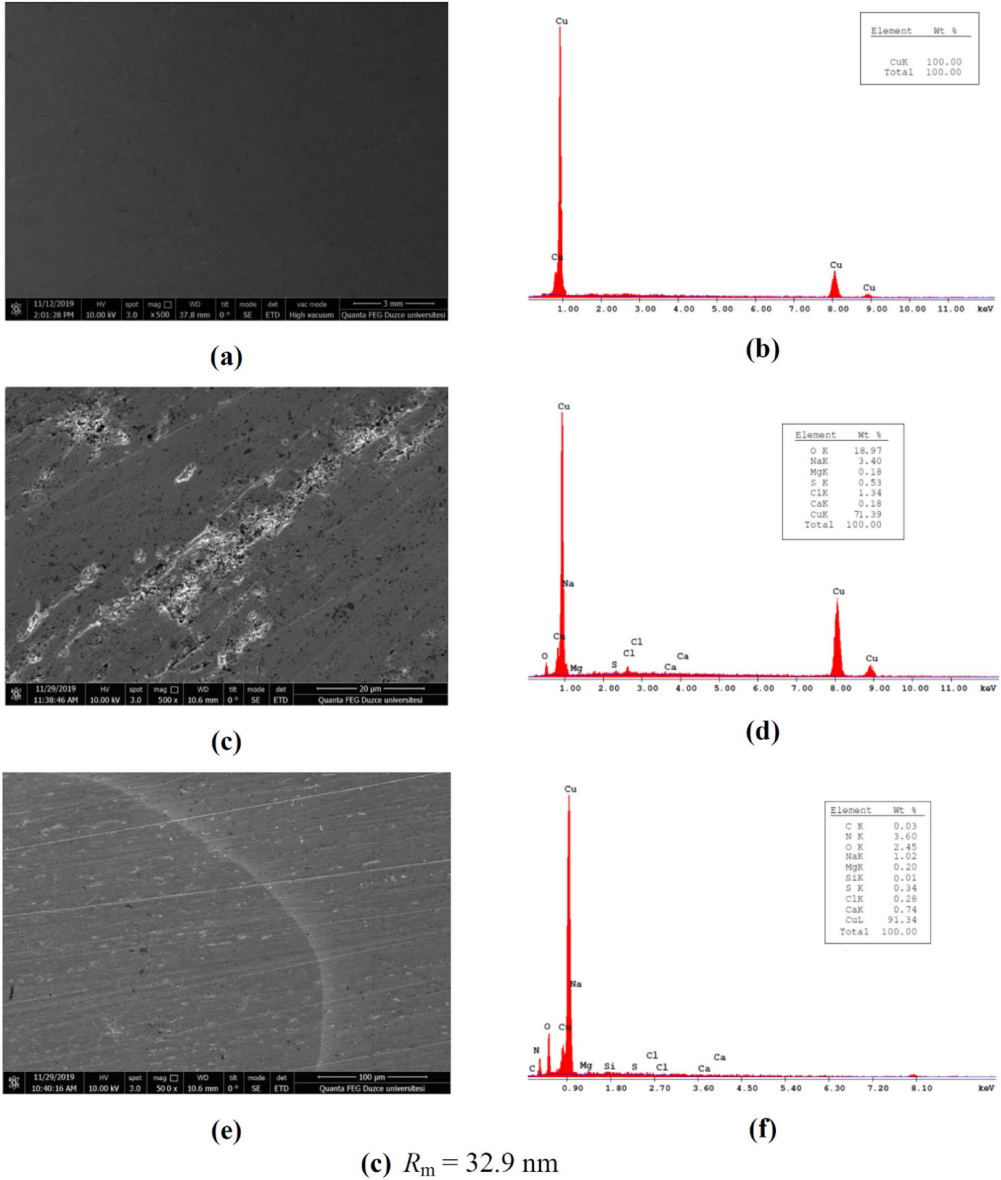
SEM and EDX images for (**a**,**b**) polished copper specimen, (**c**,**d**) copper specimen corroded in SCW and (**e**, **f**) copper specimen in SCW protected by 2000 ppm NaNO_2_.

Figure 5a,b,c,d,e and f represented the SEM and associated EDX graphs of copper specimen having different morphologies and compositions in different environments. The changes occurring on the copper surface were apparent from the images obtained by SEM and elemental composition of the surface suggested by EDX.

The SEM of polished copper in Fig. 5a displayed an un-corroded, fairly smooth surface with a few abrasion lines, which could be attributed to surface preparation by SiC paper. The relative EDX graph indicated that the surface was devoid of any corrosion products and is pure copper specimen (Fig. 5b). The SEM of the copper sample immersed in SCW in Fig. 5c displayed a considerably corroded and pitted surface which can be associated with the presence of chloride, sulphur and oxygen signals in the EDX spectra (Fig. 5d). On the surfaces of corroded specimen localized corrosion was more apparent along with some uniform corrosion. The amount of chlorides and sulfide ions on the surface was bearing a low signal because of their low millimolar concentration in SCW. However it is interesting to note that even in such concentrations these aggressive ions have posed considerable local damage to the copper surface. The oxygen signal was more apparent than the chloride signal and even its weight percentage is higher than that of chloride, which indicated the presence of oxides on the corroded surface, particularly cuprous oxide in case of uninhibited specimen.

The primary feature noticed in the SEM of the copper specimen inhibited by 2000 ppm NaNO_2_ in Fig. 5e was that considerable corrosion and pitting which was previously observed, now highly reduced compared to the uninhibited specimen corroded in SCW.

To get a more clear inference, the EDX values of the elements present on the copper substrate displayed the occurrence of considerable amounts of N, O and trace amounts of S. The amount of Cl^−^ ions was reduced in the inhibited surface according to the EDX graph (Fig. 5f) which suggests that nitrite ions have clearly prevented the association of chloride with the copper surface. The signal of oxygen was a little low in inhibited specimen because sodium nitrite is more favorable to the development of cupric oxide and hydroxides on the copper surface, which could have undergone displacement by inhibitor molecules too and hence a low signal for oxygen was obtained. The EDX spectrum also displayed an improvement in copper percentage in inhibited specimen.

#### Optical profilometry

The optical profiling was done to know the surface roughness, materials loss and pits statistics on the copper surface to understand the effect of SCW and inhibitor on it.

**Figure 6:**
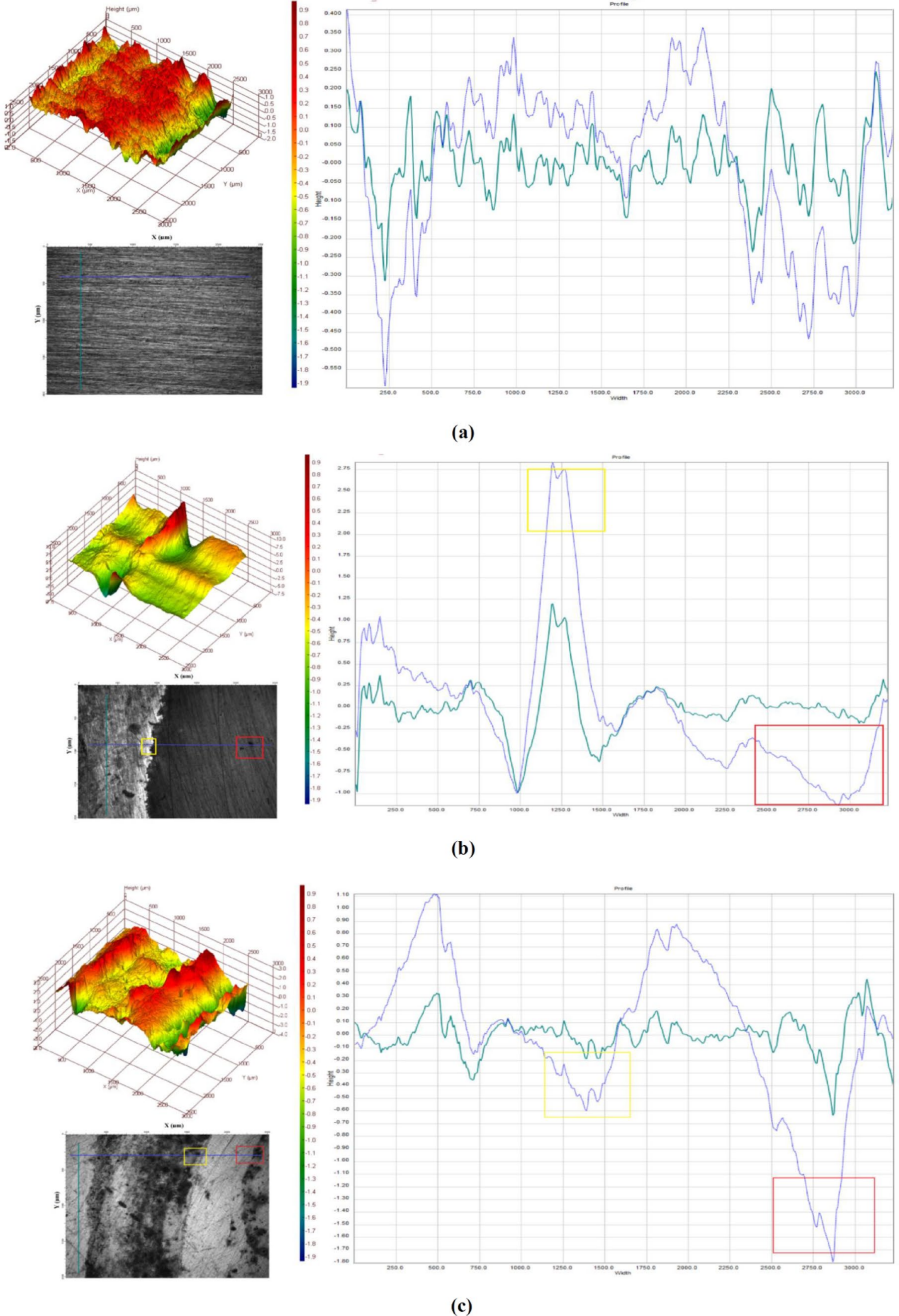
Optical profile for (**a**) polished copper specimen, (**b**) copper specimen corroded in SCW and (**c**) copper specimen in SCW protected by 2000 ppm NaNO_2_.

The 3D image very clearly depicted the red regions on the polished sample (Fig. 6a), the uncorroded red areas diminished in the uninhibited corroded specimen (Fig. 6b), while the inhibited sample showed corroded as well as more protected areas in the 3D image (Fig. 6c). The 2D image and the height profile clearly displayed the occurrence of localized and pitting corrosion in corroded specimen which was immersed in SCW (Fig. 6b). The inhibited specimen displayed an improvement in the depth of valley ratio and average roughness of the whole sample in the presence on 2000 ppm NaNO_2_ in SCW (Fig. 6c). The quantitative data is present in the Table 4.

**Table 4:** Optical profile results of copper in SCW inhibited by 2000 ppm NaNO_2_.

Specimens	Average roughness *R*_a_ (μm)	Mean texture depth *R*_t_ (μm)	Roughness depth *R*_z_ (μm)	Average valley depth *R*_v_ (μm)	Average peak height *R*_p_ (μm)
Polished	0.06	0.57	0.38	− 0.31	0.24
Blank	0.21	2.23	1.16	− 1.01	1.23
2000 ppm NaNO_2_	0.11	1.09	0.55	− 0.51	0.45

### Computational analysis

**Figure 7:**
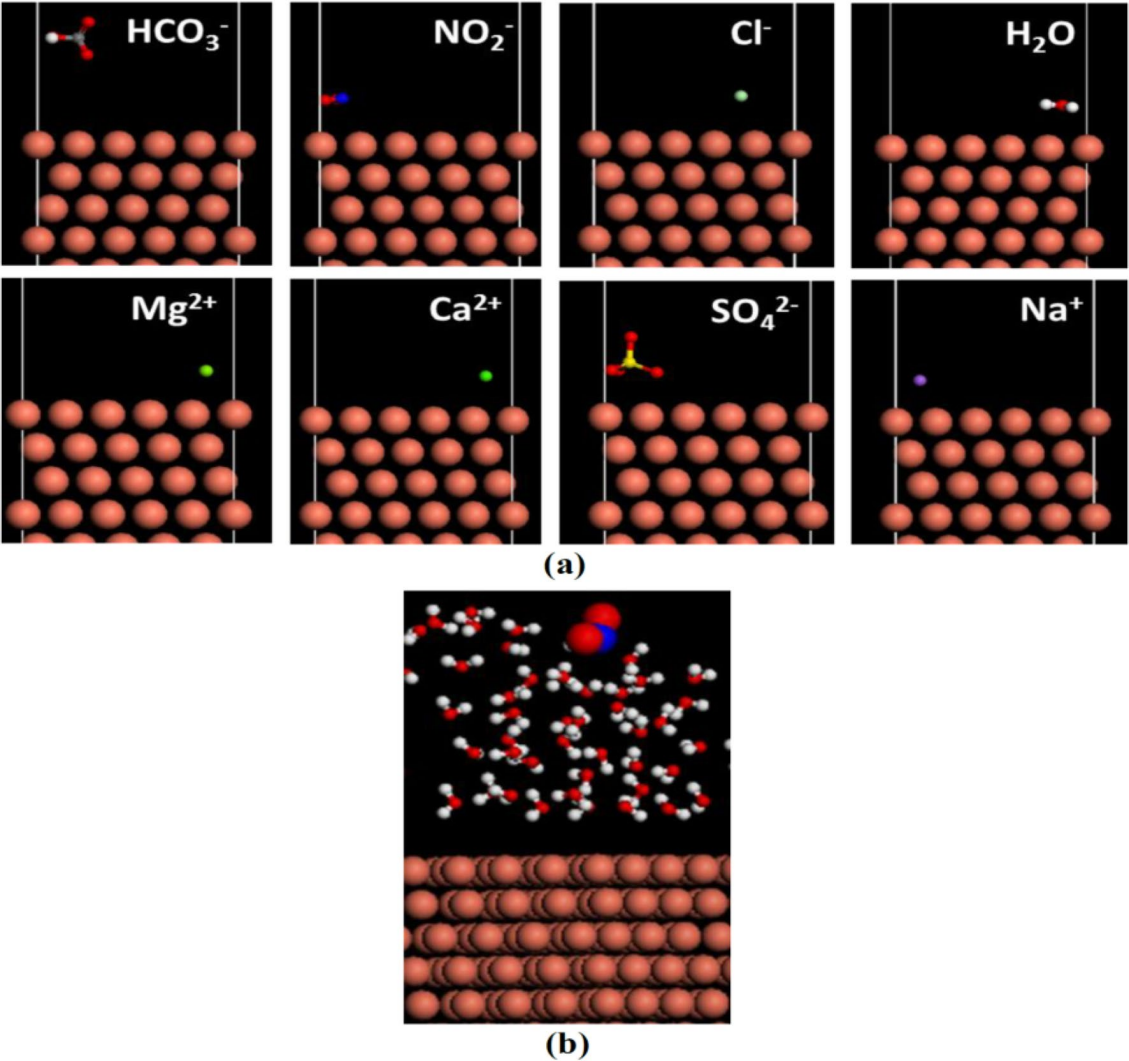
(**a**) Adsorption mode of different solution constituents onto the copper surface and (**b**) equilibrium configurations at the solution/copper interface.

In Table 5, frontier orbital energies, energy gap, electronegativity, hardness, fraction of electrons transferred, electron donating power, electron accepting power and dipole moment values calculated for nitrite ion at various calculation levels of the theory are given as detailed (Fig. 7). Frontier orbital energies, namely HOMO/high occupied molecular orbital energy and LUMO/low unoccupied molecular orbital energy, are widely considered in the analysis of corrosion inhibition performances of molecules. High values of E_HOMO_ facilitate the adsorption of inhibitor molecules on metal surfaces and therefore enhance the inhibition efficiency. On the other hand, the energy level of the lowest unoccupied molecular orbital represents the ability of the molecule to accept electrons from an electron donor. Energy gap, namely, the difference between HOMO and LUMO orbital energies is an important indicator of the reactivities of molecules. It should be noted that high energy gap values belong to the molecules with low reactivity. This situation can be explained in the light of Maximum Hardness Principle.

**Table 5:** Electronic structure parameters of nitrite ion using different methods/basis set combinations.

Method	E_total_ (eV)	E_HOMO_ (eV)	E_LUMO_ (eV)	ΔE (eV)	η (eV)	χ (eV)	ΔN_Cu(111)_	μ (D)	ω (eV)	ω^+^ (eV)	ω^−^ (eV)
DFT 6–311++G(d.p)	− 5543.866	− 5.588	− 0.522	5.067	2.533	3.055	0.372	0.530	1.842	0.631	3.686
DFT 6-311G	− 5541.207	− 4.888	− 0.274	4.614	2.307	2.581	0.511	0.352	1.443	0.441	3.022
DFT AUG-CC-PVDZ	− 5543.062	− 5.544	− 0.487	5.058	2.529	3.015	0.380	0.539	1.797	0.606	3.621
HF 6–311++G(d.p)	− 5514.858	− 9.678	3.433	13.111	6.556	3.123	0.139	0.847	0.743	3.994	10.549
HF 6-311G	− 5511.735	− 9.524	4.739	14.263	7.132	2.392	0.179	0.792	0.401	7.362	14.493
HF AUG-CC-PVDZ	− 5514.192	− 9.631	2.935	12.566	6.283	3.348	0.127	0.759	0.892	3.172	9.455
MP 2 6–311++G(d.p)	− 5530.676	− 10.033	3.445	13.479	6.739	3.294	0.122	0.976	0.805	3.935	10.674
MP 2 6-311G	− 5522.846	− 9.941	3.841	13.782	6.891	3.050	0.137	0.965	0.674	4.720	11.611
MP 2 AUG-CC-PVDZ	− 5529.915	− 10.030	2.946	12.976	6.488	3.542	0.108	0.891	0.966	3.140	9.628

According to hard and soft acid–base principle introduced by Pearson, hardness, softness and energy gap are closely related parameters to each other. Pearson defined the hardness as the resistance towards electron cloud polarization or deformation of chemical species. The hard molecules with high energy gap values are not effective against the metal corrosion. On the other hand, soft molecules with low energy gap values are good corrosion inhibitor. In the hard and soft acid base classification of Pearson, NO_2_^−^ ion appears between borderline bases. Here it is apparent that nitrite ion is more effective against the corrosion of copper surface compared to nitrate ion that is a hard base. It could be seen from the data given in Table 5 that hardness values calculated for nitrite ion are quite low in general. According to maximum hardness principle^36^ hard molecules are more stable compared to soft ones. Adsorption behavior of nitrite ion on copper surface supported the validity of this electronic structure principle. The tendency of a chemical species to attract electrons is explained with the help of electronegativity concept. The molecules having high electronegativity values are not effective against the metal corrosion. Electronegativity values calculated for studied inhibitor molecule were not that high.

Another useful parameter used in corrosion inhibition studies is electrophilicity index. An electronic structure principle known as minimum electrophilicity principle correlates the electrophilicity index with chemical stability. According to this principle the natural direction of a chemical reaction is toward a state of minimum electrophilicity. From here, it can be concluded that the molecules with low electrophilicity value are more stable. In contradiction with this principle, in corrosion studies, it is widely reported that the molecules having high electrophilicity values are not good corrosion inhibitor. Nitrite ion with low electrophilicity value is a good corrosion inhibitor for copper surface. This result shows that minimum electrophilicity principle^37^ is not successful in the explanation of corrosion inhibition activities of molecules. According to electron donating and electron accepting power parameters derived by Gazquez and coworkers, good corrosion inhibitors should have high electron donating power values. It is apparent from the related table that electron donating power values calculated for studied ion is quite high. Adsorption energy values obtained molecular dynamics simulation approach shows that the interaction between nitrite ion and copper surface is quite powerful. It can be understood from the data given in Table 6 that nitrite ion interacts more strongly with Cu (111) surface compared to other ions. Theoretically obtained results are in good agreement with experimental observations.

**Table 6:** Adsorption energy (E_ads_) of solution constituents (isolate species) and (species adsorbed) onto the copper surface.

Isolated/adsorbed	Species	E_ads_ (Kcal/mol)
Isolated	HCO_3_^–^	1.396
	NO_2_^–^	− 57.976
	Cl^–^	− 0.329
	Na^+^	− 0.089
	Mg^2+^	− 0.176
	Ca^2+^	− 0.289
	SO_4_^2–^	232.062
	H_2_O	− 1.638
Adsorbed	NO_2_^–^	− 60.535
	H_2_O	− 12.293

## Conclusions

The effect of addition of NaNO_2_ to the electrochemical and surface properties of copper in SCW was observed. The following inferences were made:

1. A maximum concentration of NaNO_2_ at 2000 ppm prevented 61.8% of corrosion as suggested by EIS. The EIS parameters suggested the formation of a thin barrier film which improved with increase in inhibitor concentration. The first inference which was derived was that nitrite ions have promoted the growth of passive oxide layer on copper surface.
2. The DEIS analysis gave some important insights into role of nitrite ion in inhibition of copper corrosion in SCW. The nitrite ion diminished the growth of initial oxide layer formation of Cu_2_O but at exactly after two hours improved the inhibition of copper when Cu_2_O + Cu(OH)_2_ was formed after 14,400 s. The inhibition effect was 50–59% over the period of 24 h. The diminishing of the IE values at 12 and 24 h could be associated to the loss of nitrite ions from the copper surface which were physiosorbed and gave a good protection (59.5%) at 6 h interval. This kind of behaviour of NaNO_2_ could not be detected and explained solely through EIS which raised the importance of DEIS in corrosion measurements. Another important inference derived from the DEIS data was that this inhibitor continued to be effective even after 24 h.
3. The adsorption studies suggested spontaneous and predominant physiosorption of sodium nitrite on copper in SCW. The adsorption studies also suggested that each active site on the copper surface was associated with more than one adsorbing species. This could be attributed to formation of either Cu(OH)_2_ or Cu(NO_2_)_2_. The studies also suggested the lesser interaction among the adsorbed species. The comprehensive type adsorption suggested by El-Awady isotherm indicated some role of chemisorption in the process too, which might be due to the formation of [Cu/Inhibitor] complex using oxygen or nitrogen.
4. The inhibitor NaNO_2_ was really effective in improving the surface homogeneity as suggested by AFM and OP analysis.
5. One of the most interesting inferences was derived from SEM/EDX analysis. The inhibition which was observed cannot be attribute solely to the formation of passive layer Cu_2_O + CuO/Cu(OH)_2._ Considerable amounts of nitrogen and oxygen detected through EDX analysis in inhibited specimen suggested an association of nitrite ion to the copper surface.
6. The parameters derived by the HOMO and LUMO energy of system suggest that nitrite ion has a low electrophilicity value and high electron donating power. The molecular dynamic simulation indicated a powerful interaction between nitrite ion and copper surface i.e. sodium nitrite might be regarded as an anodic inhibitor, but through experimentations it has been observed that it may act as film forming inhibitor for copper in simulated cooling water.
7. All the analysis performed to establish the role of sodium nitrite in protecting copper in SCW suggested that it was a very efficient inhibitor for cooling water systems fabricated with copper.

## Methods

### Materials and media

The copper samples certified to be 99.99% pure obtained from Sofıa Med Ad, Bulgaria, were utilized as test specimens. The test specimens’ working surface was abraded by SiC papers ranging from 360 to 2000 grit, degreased with alcohol and subsequently washed with distilled water. The simulated cooling water was prepared by dissolving NaCl (7.5 mmol/L), NaHCO_3_ (2.0 mmol/L), Na_2_SO_4_ (3.5 mmol/L), MgSO_4_ (0.25 mmol/L) and CaCl_2_ (0.50 mmol/L) in distilled water^38^.

### Electrochemical impedance spectroscopy (EIS)

The experiments were performed in a system where Ag/AgCl acted as reference electrode, a counter electrode of platinum (Pt) and working electrode of copper with exposed surface area 0.785 cm^2^ formed the investigation cell. The steady state OCP was obtained at interval of two hours. EIS was conducted at 10 mV amplitude signal peak-to-peak corrosion potential (*E*_corr_) with a 10 mHz to 100 kHz of frequency range^39^. The output was processed by the ZsimpWin 3.21 software. The % inhibition efficiency ‘*IE*’ values were computed according to the following equation.

11 $$IE(\% ) = \frac{{R_{ct}^{(i)} - R_{ct} }}{{R_{ct}^{(i)} }} \times 100$$

where *R*_ct_^(i)^ represents the charge transfer resistance in the presence of NaNO_2_ while *R*_ct_ is the value for uninhibited solution, respectively.

### Dynamic electrochemical impedance spectroscopy (DEIS)

The effectiveness of inhibitors against scaling and corrosion of copper over longer time periods can be monitored through DEIS. DEIS was performed using current perturbation signal given by National Instrument Ltd PCI-4461 digital-analog card and acquisition of voltage response signal of copper sample. The galvanostatic conditions were established by Slepski Galvanostat which also served as current–voltage converter during the process^40^. Multisinusoidal signal, which consisted of 20 elementary current sinusoids of optimized phase shift and amplitude value, was used as perturbation source. The sampling frequency was 12.8 kHz, and the frequency range from 300 mHz to 4.5 kHz, with 8 points per decade of frequency was applied. The DEIS assures the live assessment of the corrosion process on working electrode. The impedance spectra obtained by DEIS were analyzed with the same program that interpreted the EIS data.

### Surface analysis

Visual realization of the degree of corrosion occurring on the copper specimens’ surface in terms of heterogeneity/roughness requires high precision techniques like AFM and SEM/EDX. The current experiments were conducted on copper specimens in inhibited and uninhibited conditions at room temperature. SEM/EDX study was done by FEG250 (FEI, Holland) with EDX attachment. The instrument for AFM was ‘Park Systems XE-100E model. The scan rate was 0.2 Hz and optical resolution was 1 μm. The area of (9.09 × 9.09) μm^2^ of test coupon was visualized in non-contact mode. The optical profilometry was conducted by Phase View optik profiler.

### Computational methods

In the Conceptual Density Functional Theory introduced by Parr and Pearson, descriptors of chemical reactivity like chemical potential (µ), electronegativity (χ), hardness (η) and softness (σ) are derived with respect to number of electron of total electronic energy at a constant external potential. Softness is the multiplicative inverse of the hardness. Electronegativity is reported as the negative value of chemical potential. Within the framework of finite differences approach, the relation with ground state ionization energy (I), ground state electron affinity (A), total electronic energy E and the number of electrons (N) of the aforementioned descriptors are presented via the following equations^41^.

$$\mu = - \chi = \left[ {\frac{\partial E}{{\partial N}}} \right]_{\nu (r)} = - \left( {\frac{I + A}{2}} \right)$$

$$\eta = \frac{1}{2}\left[ {\frac{{\partial^{2} E}}{{\partial N^{2} }}} \right]_{\nu (r)} = \frac{I - A}{2}$$

$$\sigma = 1/\eta$$

Koopmans Theorem^42^ can be considered as a bridge between Molecular Orbital Theory and Conceptual Density Functional Theory. Koopmans theorem states that the negative values of HOMO and LUMO orbital energies relate to ionization energy and electron affinity of molecules is a parallel approach to predict the molecular ionization energy and electron affinity values. In the light of this theorem, the following relations $I = - E_{HOMO}$ and $A = - E_{LUMO}$ are given. According to electrophilicity index (ω) given by Parr, Szentpaly and Liu^43^, electrophilic power of chemical species can be predicted using their electronegativity (or chemical potential) and hardness values. After this well-known study, Chattaraj proposed the nucleophilicity (ε) as the multiplicative inverse of electrophilicity.

$$\omega = \chi^{2} /2\eta = \mu^{2} /2\eta$$

$$\varepsilon = 1/\omega$$

In recent years, Gazquez and coworkers^44^ made detailed studies regarding to the prediction of electron donation and electron accepting abilities of molecules and they proposed two new parameters called as electron donating power (ω^−^) and electron accepting power (ω^+^) and formulated these parameters based on ionization energy and electron affinity concepts via following equations.

$$\omega^{ + } = (I + 3A)^{2} /(16(I - A))$$

$$\omega^{ - } = (3I + A)^{2} /(16(I - A))$$

The fraction of electrons transferred (ΔN) from inhibitor molecule to metal surface is an important parameter. The equation proposed for the calculation of this parameter has been suggested considering electronegativity equalization principle imparted to science by Sanderson. Until recently, for the calculation of the fraction of electrons transferred, electronegativity of metal atom was in use but some researchers proposed the use of work function determined for metal surface instead of metal electronegativity. The equation for the calculation of ΔN values of adsorption processes is given as:

$$\Delta N = \frac{{\phi_{Cu} - \chi_{inh} }}{{2(\eta_{Cu} + \eta_{inh} )}}$$

wherein, it is important to note that χ_inh_, η_Cu_ and η_inh_ stand for electronegativity of inhibitor, hardness of metal and hardness of inhibitor, respectively. In the calculations, η_Cu_ = 0 is taken assuming that for a metallic bulk I = A. Work function value reported for Cu (111) surface is 4.80 eV^45^.

### Molecular dynamics simulation

Adsorption properties of nitrite ion (NO_2_^−^) were estimated using Molecular Dynamics Simulation Approach on Forcite module from Accelrys Inc. As model metal surface Cu (111) was selected. To see the effect of solvent, the calculations were made in both vacuum and aqueous media. The calculations were made in a simulation box where nitrite ion is in a contact with copper surface at 303 K. Molecular Dynamic simulation approach is well established for the analysis of metal-inhibitor interaction. It is notably important that in the calculation of interaction (E_interaction_) and binding (E_binding_) energies regarding to adsorption process of nitrite ion on Cu (111) surface, the following equations are considered^46^.

$$E_{{{\text{interaction}}}} = E_{total} - (E_{{surface + H_{2} O}} + E_{inhibitor} )$$

$$E_{binding} = - E_{{{\text{interaction}}}}$$

In the given equations, E_total_ stands for the total energy of all system. E_surface+H2O_ represent the total energy of Cu (111) surface with H_2_O molecules. E_inhibitor_ is the total energy of inhibitor alone.
