# Author Correction: Sodium nitrite as a corrosion inhibitor of copper in simulated cooling water

**DOI:** 10.1038/s41598-021-95574-7

**Published:** 2021-07-29

**Authors:** Marziya Rizvi, Husnu Gerengi, Savas Kaya, Ilyas Uygur, Mesut Yıldız, Ibrahim Sarıoglu, Zafer Cingiz, Michal Mielniczek, Brahim El Ibrahimi

**Affiliations:** 1 Corrosion Research Laboratory, Department of Mechanical Engineering, Faculty of Engineering, Duzce University, Duzce, Turkey; 2 Department of Pharmacy, Health Services Vocational School, Sivas Cumhuriyet University, 58140 Sivas, Turkey; 3 Department of Electricity and Energy, Duzce Vocational High School, Duzce University, Duzce, Turkey; 4 Corrosion and Materials Engineering, Department of Electrochemistry, Faculty of Chemistry, Gdansk University of Technology, 11/12 Narutowicza, 80‑233 Gdańsk, Poland; 5 Applied Chemistry‑Physics Team, Materials and Environment Laboratory, Faculty of Sciences, Ibn Zohr University, B.P. 8106, Cité Dakhla, Agadir Morocco

Correction to: *Scientific Reports* 10.1038/s41598-021-87858-9, published online 16 April 2021

The original version of this Article contained an error, where unit “mHz” was incorrectly given as “MHz”.

As a result, in the Methods section, under the subheading ‘Electrochemical impedance spectroscopy (EIS)’,

“EIS was conducted at 10 mV amplitude signal peak-to-peak corrosion potential (Ecorr) with a 10 MHz to 100 kHz of frequency range^39^”.

now reads:

“EIS was conducted at 10 mV amplitude signal peak-to-peak corrosion potential (Ecorr) with a 10 mHz to 100 kHz of frequency range^39^”.

And under the subheading ‘Dynamic electrochemical impedance spectroscopy (DEIS)’,

“The sampling frequency was 12.8 kHz, and the frequency range from 300 MHz to 4.5 kHz, with 8 points per decade of frequency was applied”.

now reads:

“The sampling frequency was 12.8 kHz, and the frequency range from 300 mHz to 4.5 kHz, with 8 points per decade of frequency was applied”.

The original Article has been corrected.

